# Looking beyond the cancer cell for effective drug combinations

**DOI:** 10.1186/s13073-016-0379-8

**Published:** 2016-11-25

**Authors:** Jonathan R. Dry, Mi Yang, Julio Saez-Rodriguez

**Affiliations:** 1 Oncology Innovative Medicines and Early Development, AstraZeneca, R&D Boston, Waltham, MA 02451 USA; 2 European Molecular Biology Laboratory, European Bioinformatics Institute (EMBL-EBI), Wellcome Genome Campus, Cambridge, CB10 1SD UK; 3 Rheinisch-Westfälische Technische Hochschule Aachen University, Faculty of Medicine, Joint Research Centre for Computational Biomedicine, Aachen, 52057 Germany

## Abstract

Combinations of therapies are being actively pursued to expand therapeutic options and deal with cancer’s pervasive resistance to treatment. Research efforts to discover effective combination treatments have focused on drugs targeting intracellular processes of the cancer cells and in particular on small molecules that target aberrant kinases. Accordingly, most of the computational methods used to study, predict, and develop drug combinations concentrate on these modes of action and signaling processes within the cancer cell. This focus on the cancer cell overlooks significant opportunities to tackle other components of tumor biology that may offer greater potential for improving patient survival. Many alternative strategies have been developed to combat cancer; for example, targeting different cancer cellular processes such as epigenetic control; modulating stromal cells that interact with the tumor; strengthening physical barriers that confine tumor growth; boosting the immune system to attack tumor cells; and even regulating the microbiome to support antitumor responses. We suggest that to fully exploit these treatment modalities using effective drug combinations it is necessary to develop multiscale computational approaches that take into account the full complexity underlying the biology of a tumor, its microenvironment, and a patient’s response to the drugs. In this Opinion article, we discuss preliminary work in this area and the needs—in terms of both computational and data requirements—that will truly empower such combinations.

## Background

Advances in tumor profiling and deep sequencing have revealed driver mutations and yielded novel targets for a new generation of cancer drugs. Despite progress in our abilities to determine and diagnose genetically defined tumor subgroups and patients most likely to benefit from available treatments, these therapies have yet to realize their full potential, owing in part to the intrinsic and adaptive resistance of tumors [1]. Within cancer cells, compensatory signaling pathways can be harnessed to overcome a dependency on any one drug target. This plasticity of tumor cells enables dedifferentiation and avoidance of cell death. Furthermore, inherent DNA instability leads to extensive heterogeneity and rapid clonal evolution of tumor cells.

A simple literature search reveals hundreds of examples of both experimental and computational approaches that have been used to discover pairs of drugs that may offer enhanced benefit if used in combination to treat cancer [2–4]. Owing to their in vitro nature, most experimental phenotypic screens search for pairs of drugs that act synergistically to increase growth inhibition or induce death of specific cancer cells [5–7]. Similarly, many computational methods focus on the identification of drug cocktails to enhance effects that are specific to the cancer cell by increasing the degree to which intracellular oncogenic bioactivity is suppressed [4, 8, 9]. Both these approaches are based on the principle that by hitting the cancer cell “harder and faster” the tumor response will be more dramatic and the likelihood of cells escaping and resistance emerging will be reduced. Although these approaches can be effective, the focus on the cancer cell overlooks the considerable opportunities for combination therapies to exploit targets outside the tumor cell.

In this Opinion article we highlight the breadth of opportunities that are available to improve the longevity of therapeutic benefit by targeting components of tumor biology such as the microenvironment or immune response in combination with tumor-cell-targeting agents. To date, hypothesis-free discovery of such multimodal drug combinations has been impractical owing to the diversity of possibilities, the variability of cellular and molecular contexts, the practicality of preclinical modeling, the paucity of data available, and the complexity of associated computational modeling [2, 10]. We outline new technologies and advocate the collection and sharing of clinical and laboratory data necessary to enable computational prediction of testable multimodal drug combination hypotheses. In addition, we argue for the development of novel approaches that can model such multiscale combined phenomena and assess the likelihood that resulting drug combinations will achieve clinical benefit.

## Potential benefit from drug combinations with targets outside the primary tumor cell

Successful drug combinations used in clinical practice today, and those emerging in current clinical trials, indicate that more attention should be given to targets outside the tumor cell. Of the 521 non-small-cell lung carcinoma (NSCLC) drug combination trials that have been completed for which an outcome is reported in Trialtrove [11], 184 combine multiple drugs that have targets inside the tumor cell, whereas 110 trials combine such tumor-cell-targeting drugs with angiogenic agents and 94 with immune-targeting agents (Box 1). Many clinical drug combination successes seem to involve drug pairs with independent effects rather than synergistic activity within the tumor cell [12, 13]. Furthermore, the considerable increase in immunotherapies in recent years is apparent among published and ongoing combination trials (Box 1). It is important, therefore, to discover additional combination approaches that consider all aspects of biology in patients with cancer to best improve responses by both controlling the tumor and improving patient wellness, while avoiding antagonism and toxicity.

### Targeting independent subpopulations of cancer cells across heterogeneous tumors

Tumors have inherent DNA instability and encounter sequential environmental and therapeutic selective pressures throughout their development. In addition, migration and metastasis lead to the independent evolution of tumor cell populations at distal sites in diverse environmental conditions. As a result, an advanced cancer can comprise multiple subclonal tumors, each with independent genetic drivers and responses to particular therapies [14, 15]. Most therapeutic choices concentrate on the driver events that are most prevalent across the primary tumor; however, recurrence can result from the outgrowth of small pre-existing resistant cell populations [16]. Drug combination approaches designed to tackle several independent drivers offer great promise, in particular to combat subclonal populations that are likely to be resistant to the primary therapy [17]. Another attractive approach is to control tumors by using sequential, tailored therapy that is informed by continuous monitoring of tumor evolution. Such therapy could be adapted following the detection of clonal outgrowth to maximize therapeutic benefit; this approach to therapy is referred to as “temporal collateral sensitivity” [16, 18, 19].

In addition to inherent heterogeneity and subclonality, epigenetically driven changes in cell state can give rise to dedifferentiated cell populations that survive many therapeutic pressures and have a pivotal role in the development of resistant tumor-cell populations [15, 20]. Combination therapies aimed at reducing the plasticity of tumor cells, synchronizing the cell cycle or otherwise maintaining sensitized tumor cell states, or targeting epigenetic dysregulation hold additional promise for the prevention of drug resistance and tumor evolution [3, 21, 22]. For example, enhanced or prolonged tumor responses have been reported using drug combinations that inhibit DNA repair to sensitize tumor cells to DNA-damaging agents [23]; target epigenetic regulators to prevent cell state transition [15]; or synchronize the cells’ DNA repair cycle at a point that is sensitive to chemotherapy [24].

### Improving response rates by identifying drugs with independent non-antagonistic effects

Despite advances in companion diagnostics (that is, tests for biomarkers associated with enhanced response to a particular drug) and precision medicine (that is, biomarker-led tailoring of therapies to an individual patient), patient selection remains imperfect, and most marketed agents have suboptimal response rates in their prescribed indications [5, 22]. Objective response rates in successful oncology drug trials are typically below 40% and are not significantly higher than those in many failed trials (Trialtrove) [11], which suggests that many therapies may fail in early trials because of a lack of improved response rate in a defined population for which response to any drug is infrequent. Rarely, however, is it proven whether the population in a failed trial is distinct from the population responding to the comparator or standard of care therapy. It may therefore be pertinent to pay more attention to drugs that benefit different patients across a clinically or molecularly defined population without antagonism or significant adverse events.

### Targeting tumor promotion and protection conferred by the stroma and extracellular matrix

**Fig. 1:**
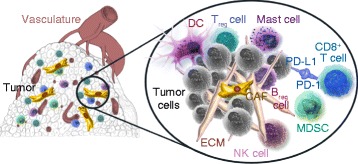
The tumor microenvironment. Many cells and tissue components interact with cancer cells to influence tumor progression and response. These include cytotoxic (CD8^+^) T cells and regulatory T (T_reg_) cells, regulatory B cells (B_reg_), dendritic cells (DCs), natural killer (NK) cells, myeloid-derived suppressor cells (MDSCs), and mast cells, which are involved in the immune response against the tumor and communicate with tumor cells through receptor–ligand interactions such as those between programmed cell death protein 1 (PD-1) and PD-1 ligand 1 (PD-L1). In addition, the extracellular matrix (ECM), cancer-associated fibroblasts (CAFs), and endothelial cells of the vasculature are critical to tumor growth, transformation, and angiogenesis. In addition to targeting the tumor itself, all of the described components of the tumor microenvironment represent potential therapeutic targets. Figure produced with permission of Acerta Pharma and copyright is reserved

Tumors actively remodel their microenvironment, which comprises a heterogeneous collection of endothelial cells, leukocytes, cancer-associated fibroblasts (CAFs), mesenchymal stromal cells, growth factors, proteases, and the extracellular matrix (ECM) [13, 25, 26] (Fig. 1). Chemotherapies, surgery, and radiotherapy can also influence the microenvironment, creating general tissue damage that triggers a wound-healing response and the influx of inflammatory cells [25]. The resulting microenvironment in turn promotes tumor growth and survival by influencing cell migration, differentiation, immune responses, and inflammation and protects the tumor from the effects of therapeutics [13]. Growth factors and endocrine signals delivered to the tumor from or through the microenvironment offer obvious targets for combination therapies and a number of successful therapies target these molecules [27, 28]. Development of therapies that target components of the tumor microenvironment can be complex, as many components have a critical role in normal tissues and processes, as well as in tumor control [29]. Tumor immunotherapy will be discussed separately, but other therapeutic approaches showing promise include: regulation and degradation of the ECM with matrix metalloproteinases [29]; collagenases [30]; endocrine therapies [27]; restricting vascularization with anti-angiogenics such as bevacizumab [25, 30, 31]; and manipulating the migration and functions of CAFs [32].

### Targeting the physical barrier of the tumor microenvironment

As well as its role in tumor promotion, the tumor microenvironment can physically affect tumor progression and response. Abnormal tumor vasculature, ECM, and interstitial fluid pressures (which affect blood perfusion and molecular movement) can prevent drugs from reaching tumor cells (Fig. 1). A number of therapeutic approaches are being used to shape the tumor microenvironment specifically to improve delivery of antitumor agents. These approaches include promoting or normalizing vascularization, alleviating solid stress, normalizing lymph flow, altering the composition of the tumor stroma, and elevating blood pressure [30, 33, 34]. Aside from drug-delivery considerations, however, the tumor stroma and ECM have also been reported to physically restrain tumor growth [35], a role that could potentially be therapeutically promoted.

### Promoting an antitumor response from the immune system

The success of agents that stimulate an antitumor immune response has been well documented in recent years [12, 36, 37]. The use of these agents has led to dramatic tumor responses and improved survival in a subset of patients with melanoma, and immunotherapies are showing promise in various other tumor types [38]. Multiple studies are also underway that combine immunotherapies with numerous targeted agents or cytotoxic therapies [39]; 11 such trials have been reported since 2005 whereas only one such trial was reported prior to 2005 (Trialtrove) [11].

When seeking beneficial combinations it is important to understand the potentially conflicting effects that a drug may have on the various cell types in the body. Agents designed to target tumor-cell-driving oncogenes, for example, may also impact immunosuppressive signals from the tumor cell or key signaling processes within immune cells. Numerous well-studied cancer-cell drug targets have essential roles in the normal development, differentiation, and activity of certain immune cells (Fig. 1). In tumor cells, inhibition of the mitogen-activated protein kinase kinase MEK1 (also known as MAP2K1) can dramatically arrest growth, and high expression of the immunosupressive programmed cell death protein 1 ligand 1 (PD-L1) has been associated with resistance [40]. Within immune cells, MEK1 can play contradictory roles; for example, it promotes both CD8^+^ T-cell priming and CD8^+^ T-cell death [41], which makes it difficult to predict whether inhibition would have an immunosuppressive or immunostimulatory effect (Fig. 1). Similarly, inhibition of the kinase mammalian target of rapamycin (mTOR) inhibits tumor cell growth by inducing apoptosis [42] and also reduces PD-L1 expression by the tumor cell [43]. However, mTOR inhibition also suppresses the immune response against the tumor by increasing the activity of phosphatidylinositol-3,4,5-trisphosphate (PtdIns(3,4,5)P_3_)-mediated signaling pathways in cytotoxic CD8^+^ T cells [44]. Inhibition of vascular endothelial growth factor (VEGF) may, alongside its well-studied anti-angiogenic effects, promote T-cell effector function and trafficking to the tumor, decrease PD-1 expression on CD8^+^ T cells, increase the number of immature dendritic cells and their T-cell-priming ability, and reduce the size of T-cell-regulatory myeloid-derived suppressor cell populations [45]. Indeed, preclinical and translational data have supported synergy between angiogenesis inhibitors and immunotherapies and led to multiple trials of such combinations with some early signs of success [46, 47]. As drug targets in the tumor, the microenvironment, and immune cells can have both antitumor and protumor effects, predicting the overall efficacy of targeted therapies is difficult, but a better understanding of this complex biology promises to improve predictions and also reveal the most effective ways to combine drugs.

Multiple reports now show that there is an improved antitumor immune response to cancer cells that have higher mutational loads and resulting increased antigenic burden [48]. A number of agents designed to selectively introduce DNA damage to tumor cells [23] are also now showing success in clinical practice. Although primarily designed to introduce intolerable levels of DNA damage to kill the tumor cell directly, such agents could potentially increase the antigenic burden of a tumor cell [49]. Furthermore, increased tumor cell death can lead to the release and recognition of tumor-cell-specific antigens by the immune system [39]. Such attempts to enhance the immunogenicity of cancer cells—that is, the extent to which they are detected by the immune system—could also have a role in effective drug combinations.

### Boosting tumor response by targeting the microbiome

The microbiome may have diverse roles in tumor development and response, most notably in metabolic modulation of the antitumor immune response [50, 51], antigenic priming [52, 53], and the equilibrium of immune cell populations at the tumor site [53]. Approaches to selectively enrich for beneficial microbial populations are therefore attractive options to consider in combination with tumor-cell-targeting agents. Indeed, recent literature has highlighted the potential to use antibiotics or probiotics and dietary approaches to enhance responses to cancer immunotherapies [53, 54]. These recent studies have led to the development of so-called oncomicrobiotics, which indirectly promote beneficial immune responses through optimization of the gut microbiome [55].

### Improving wellness to extend the benefits of cancer-targeting therapies

At all stages of drug development, discontinuation of therapy in patients with cancer is often unrelated to the primary efficacy endpoint. For example, more than 20% of phase II, III, or IV trials of combination therapies in patients with NSCLC listed as terminated in Trialtrove ceased owing to safety concerns or adverse effects (Box 1) and a number of the remaining trials that were terminated owing to the primary endpoint not being met and lack of efficacy may have tested tolerated dose thresholds that were too low for efficacy.

Alongside therapy to improve antitumor effects, it is therefore essential to consider the right combinations of treatments to improve overall patient wellness, tackle comorbidities, and reduce adverse events. Steroid treatment can prevent intolerable gastrointestinal toxicity and enable increased dosing of anticancer agents [56, 57]. Secondary management of chronic obstructive pulmonary disease in patients with lung cancer can prevent health deterioration and prolong administration of anticancer agents [58]. Creative intermittent scheduling and switching of therapies can help to avoid toxicities while maintaining pharmacodynamically effective doses [3, 22]. The advent of biosensors and other advanced technologies for real-time monitoring [58–60] offers an unprecedented opportunity to manage a patient’s wellness throughout their cancer care and maximize therapeutic efforts.

Much focus has been given to combinations that aim to enhance tumor cell death and objective responses, yet tumors influence and are under the influence of many components of their microenvironment (Fig. 1), and patient response is in turn influenced by much broader components of overall health. Despite advances, preclinical models are severely limited in their ability to recapitulate all facets of tumor and patient biology. It is essential, therefore, that we find complementary computational approaches to identify potential combination therapies that have a clear cellular and mechanistic rationale, and that we select the most appropriate tumor models and patients in which to test them.

## Which computational approaches can identify these multiscale modes of action?

Myriads of computational methods have been developed to analyze tumors and their interactions with therapies. Data-driven methods mine existing data in a hypothesis-free manner to identify associations that implicate certain cellular processes, which can then be followed up in more detail. These methods use the data alone or combine the data with some prior knowledge, such as information on biological pathways, to distill knowledge with more mechanistic content. Data-driven methods are typically based on a statistical or machine-learning model that predicts an outcome (for example, drug synergy) from input features (for example, properties of the tumor and of the therapies to be used), and they can be extended to consider context and conditional specificities of therapeutic effects. By contrast, knowledge-driven methods use predictive models built from our understanding of a drug’s mode of action. These methods have the power to predict the effects of drugs in specific conditions in a mechanistic and dynamic context, although they are only able to do so for drugs with modes of action that are included in the model.

### Data-driven approaches

As described above, there is an increasing interest in combining drugs with fundamentally different modes of action. To analyze and eventually predict these combinations computationally, we need methods that are able to integrate different biological processes. Such integration is probably most straightforward conceptually for pure data-driven statistical and machine-learning models. These models can handle heterogeneous types of data; in the context of predictions of combinations of targeted therapies, models can be built that integrate genomic data from cells, chemoinformatic properties of drugs, and mechanistic information on the pathways in which the drugs’ targets are embedded (for example, [4, 8, 61, 62]). Similarly, researchers could build models that combine information on chemotherapies with information on immunotherapies. However, these approaches have two main limitations: (1) the algorithms need to be trained with a large amount of data to be predictive; and (2) the resulting models provide limited mechanistic insight.

Of course any data-driven computational approach can only be as good as the data that are available to train it (Table 1). High-throughput preclinical screens have started to provide a large amount of data for combinations of targeted therapies [6, 7], but such large-scale data sets are not easy to generate. Screens in cancer cell lines are limited to modeling the intracellular effects of drugs, but efforts to measure the molecular impact of individual drugs on these cells [63] should be extended to cover more drug combinations. Cell screens should also be expanded to include cell types other than cancer cells, such as stromal cells; only a few examples of such screens are available to date [28]. Advances in organoid and ex vivo technologies present opportunities to include data about stromal cell–cell interactions and environmental plasticity in data-driven models [64, 65]. It is also important to expand in vivo screens, particularly in patient-derived tumor xenografts, to determine the effects of drugs on components of the tumor microenvironment [66]. In vivo models could also be improved by using syngeneic (that is, allograft) and genetically engineered mouse models to study interactions between immune cells and tumor cells [67].

**Table 1:** Preclinical and patient data necessary to model drug combination effects across the tumor microenvironment^a^

	Data type	Advantages	Limitations	Recommendations
Pre-clinical	Cancer cell-line drug screens	- Cost-effective route to generate a significant amount of data - Detailed data from many cell lines is available, for example, GDSC [93] and CCLE [94]	- Limited to only modeling intracellular effects - Cell culture process affects important biology - Limited to testing existing drug targets	- Obtain more data for drug combinations - Use mixed cell assays to model the roles of other cells in the tumor and microenvironment - Develop better models of diverse mechanisms (for example, longer assays and different endpoints)
	Functional genomic screens (using siRNA, CRISPR, and mutagenesis)	- No limit to the number of combinations of targets testable - Synthetic lethalities can be readily identified	- Limited to intracellular mechanisms - Focus on loss of function - Only work in a limited number of cell backgrounds	- Use a broader range of cell contexts (including non-cancer cells) - Develop gain-of-function screens - Set up repositories that enable data to be shared and publicly accessible
	Drug or target perturbation screens (post- treatment functional data)	- Provide information about a drug or target’s mechanistic impact and provide pharmacodynamics maps that are likely to be relevant across cell types - Large data sets are publicly available, for example, connectivity map and LINCS [63, 95]	- Typically focus on a few cancer cell types and/or global disease processes - Only a few small in vivo screens are available - Typically provide data on monotherapy only	- Obtain more data from non-tumor cell types involved in tumor biology - Carry out larger in vivo screens and/or meta-analyses - Acquire more data for drug combinations
	Organoids (three-dimensional buds) or ex vivo screens	Can be used to obtain data about cell–cell interactions (for example, interactions between tumor cells and cells in the microenvironment) and about environmental plasticity	Few established and/or reproducible models	Develop standards to identify non-typical phenotypic parameters that are relevant to the effects of a drug on the tumor microenvironments, for example, cell-type-specific effects and cell–cell communication
	Patient-derived tumor xenograft screens	Can model the effects of drugs on components of the tumor microenvironment	- Do not model immune interactions - Cost and ethical considerations need to be take into account when using as a discovery (versus test) tool	
	In vivo screens in GEM, syngeneic, or humanized models	Can model immune interactions	Cost and ethical considerations need to be taken into account when used as a discovery (versus test) tool	
Patient	Electronic health records	Provide information about environmental exposures, immunological and metabolic measures, diagnostic assays, comorbidities and wellness, and longitudinal follow-up data	- Key data are split across isolated records in primary care and specialist hospitals, claims systems, assay providers, and others - There are currently no curation or digitization standards	- Address data confidentiality (for example, use honest brokers) and connect disparate records for patients - Improve curation and standardization
	Deconvoluting failed trials	Necessary to follow up from failed drug trials that may overlook responding populations that are mutually exclusive	Investment is rarely available to generate and mine data from failed trials	- Use a retrospective approval route in which the responding population is shown to be distinct from the comparator or standard of care
	Profiling of cell types from healthy individuals	Projects are large and well-funded, for example, GTEx [96] and the Human Protein Atlas [97]	- Public references may not capture interpatient (or disease-influenced) variability - Findings are often assessed separately from efficacy data	- Improve integrative analyses across different types of patient data - Agree on critical measures that should be assessed for the tumor microenvironment, drug toxicity, and patient comorbidities and wellness
	Comprehensive profiling of tumor genetics and heterogeneity	Projects are large, well-funded, and include tens of thousands of tumors, for example, projects by TCGA [98] and the ICGC [99]	- Exploratory NGS is not routine for patients - Public efforts are typically diagnostic, focus on the primary tumor, use purified tumor cell content, and have low sequencing depth	- Perform more and deeper spatial single-cell profiling of longitudinal and metastatic samples - Increase multi-omic profiling of samples
	Longitudinal and metastatic tumor genomic profiles	Obtaining information about genetic shift after therapy could dramatically change our understanding of tumor drivers and heterogeneity	- Currently only a limited amount of such data is available - Ethical and practical considerations regarding the necessary invasive sampling procedures need to be taken into account	Continue to advance non-invasive monitoring approaches
	Single-cell sequencing	Provide unprecedented high-resolution information about genotypic and phenotypic heterogeneity of tumors and the tumor microenvironment, including information about cell differentiation and the effects of drugs	- Current technologies require fresh tissue biopsies and obtaining these is often impractical - Mature approaches are limited to RNA profiling	- Set up central repositories for single-cell omics data from patients and models - Advance technology that enables exploration of molecules other than RNA and of non-fresh tissue samples
	Germline genetic variation	Provide information about a patient’s inherent immunological and metabolic competencies, susceptibility to adverse events, and other aspects of wellness	Information is rarely available for patients with cancer as it is removed to avoid the risk of patients being identified and confidentiality being breached	Add functional germline variant information to public databases of tumor genetics
	Biosensors and smart wearables	Enable real-time reactive adaptation of therapy to manage response, health, and adverse events	A limited number of devices are currently available and few molecular measures are currently possible	Develop technologies to identify the most important and relevant measures
Know- ledge	Pathway and interaction networks	Able to link drug target to biology	- Typically focus only on intracellular pathways and interactions - Typically limited to protein–protein interactions	Acquire more information about the cell-context specificity of interactions and about cell–cell communication
	Regulomes	Provide omics information that is indicative of active processes	- Often miss cell-context specificity of regulomes - Focus on intracellular processes - Public resources focus on the transcriptome	- Obtain more data on cell-type-specific regulomes (as in, for example, ENCODE [100]) and on extracellular communication regulomes

*CCLE* Cancer Cell Line Encyclopedia, *CRISPR* clustered regularly interspaced short palindromic repeats, *ENCODE* The Encyclopedia of DNA Elements, *GDSC* Genomics of Drug Sensitivity in Cancer, *GEM* genetically engineered mouse, *GTEx* Genotype-Tissue Expression Project, *ICGC* International Cancer Genome Consortium, *LINCS* Library of Network-Based Cellular Signatures, *NGS* next-generation sequencing, *siRNA* small interfering RNA, *TCGA* The Cancer Genome Atlas

^a^Key pieces of preclinical and patient data that need to be generated, collected, and shared to achieve the computational ambition of modeling/predicting multimodal combination effects encompassing ECM, immune, angiogenic, and stromal components of tumor biology

It is conceivable that in the not so distant future there will be enough data at the patient level to generate statistical models that take into account multiple modes of drug action and the many pathways involved in tumor biology. In recent years, there have been efforts to integrate disparate sources of information about patients [60], multiple layers of which can be important when considering the potential benefits of combination therapy. The improved availability of longitudinal tumor genomic profiles (that is, those obtained over a long period of time to enable the selective pressures of therapy to be monitored) and metastatic tumor genomic profiles could dramatically change our understanding of tumor drivers and heterogeneity [17], but this will require continued improvements in non-invasive monitoring approaches [16, 22]. Advances in single-cell sequencing can provide unprecedented high-resolution information about the impact of drugs on different cell types and the resulting variability in cell phenotypes [14, 15]. Data about germline genetic variation are rarely available for patients with cancer and, along with historical electronic medical records, could reveal immunological and metabolic competencies, comorbidities, liabilities to adverse events, and other aspects of wellness [58–60, 68]. The new age of biosensors and smart wearables should be embraced to enable real-time adaptation of therapy to effectively manage patient response, health, and adverse events [56]. In summary, improved collection and sharing of data that are often overlooked in current assays can bring significant reward, and emerging technologies offer opportunities for new types of data to be collected at unprecedented breadth and depth (Table 1).

This deluge of data will require smart and efficient algorithms to deal with confounding factors and limited statistical power and extract the inherent relatedness and redundancies from different types of data. Machine learning is progressing rapidly to deal with large data sets, in particular via deep-learning approaches that use multilayered models [69]. Data fusion strategies enable the integration of information about a common phenomenon from different detectors, so that new information can be derived by comparing the analysis of the combined data sets with separate analyses of each data set alone [70]. As the different types of data provide complementary but incomplete information (Table 1), approaches such as data-driven approaches that can integrate and combine heterogeneous types of data are likely to be essential.

Also instrumental will be text-mining approaches. The potential to advance research, therapy, and disease management by simply gaining a better grasp of the vast amount of knowledge that is already available from literature, databases, health records, and the internet has attracted efforts in the life sciences field from commercial entities such as IBM’s Watson [71] and stimulated innovation challenges that aim to develop artificial intelligence such as those planned by the US Defense Advanced Research Projects Agency [72]. The knowledge framework that these algorithms will build should provide a scaffold on which advanced machine-learning methods and information theory can discover and rationalize trends that might otherwise have been missed.

### Knowledge-driven approaches

In contrast to data-mining approaches, dynamic models that describe the clinical action of therapies at the organism level provide the basis for pharmacokinetic and pharmacodynamic studies. These dynamic models are instrumental in the development of therapies and their use in the clinic but include very limited mechanistic detail and are typically focused on preconceived hypotheses. Such models can be adapted to analyze key challenges of cancer treatment, such as resistance mechanisms, that can be modeled jointly with population-level patient survival data [73]. They can be applied to study drug combinations, and efforts have been made to jointly consider therapies with different modes of action, including chemotherapies and vascular agents [74] or chemotherapies and immunotherapies [75]. These models, however, describe the modes of action in a simple and phenomenological way. To truly integrate molecular data, such as the increasingly available genomic data from patients, we need to combine the biochemical underpinnings of the modes of action of drugs with physiological pharmacodynamics, typically in the form of ordinary differential equations, to generate so-called enhanced pharmacodynamic models [76]. This combination of pharmacokinetics and pharmacodynamics (PK/PD) with mechanistic modeling is the aim of the emerging field of quantitative and systems pharmacology [77, 78].

Combinations of small-molecule inhibitors or biologics that target signaling receptors can be analyzed through mechanistic models of the downstream signaling networks as, for example, logic circuits, causal networks, or differential equations describing the underlying biochemical reactions [79–83]. However, if we want to consider a combination of a small-molecule inhibitor that targets a kinase and a drug that affects metabolism or gene regulation, we would need integrated models of both molecular layers. Although these molecular layers have been modeled mechanistically in detail in isolation, approaches that include both layers are sparse [84–86].

The challenge of building a model that includes the modes of action of various therapies increases dramatically if we want to include therapies that affect processes occurring outside the cancer cell. For example, to investigate the interplay among tumor cells, immune cells, and angiogenesis we would require models that incorporate the intracellular molecular processes affected by the drugs in each of the relevant cell types and we would then need to combine this information in a cell–cell communication model. This approach takes into account only the effects of treatment (pharmacodynamics); modeling also the pharmacokinetics (how the organism deals with the therapy) adds yet another level of complexity, particularly as in this case one drug targets the vasculature responsible for delivering the drug and the immune cells to the tumor. A multiscale approach is required to take into account all the molecular, cellular, and physiological layers of processes occurring in an organism with cancer, including the effects of drugs and of the organism’s own immune system [87].

Which approaches enable the generation of such multiscale models and at what point do they become useful for prediction? If the different aspects are to be considered in a dynamic and quantitative manner, such as when modeled with differential equations, the model becomes very large and complex and requires an amount of information and data that is not typically available or practical, unless most of the molecular detail is sacrificed. Simpler formalisms than biochemistry-based differential equations may provide a way forward. In particular, logic modeling (also known as logical modeling) has been applied in diverse contexts that have relevance for cancer therapies, from the main apoptotic and mitogenic pathways in tumor cells to the cell cycle and cell–cell communication [88, 89]. In a logic model both molecular and phenomenological relationships can be encoded in the same formalism, enabling the inclusion of different layers, such that signaling pathways can be connected to downstream phenotypes to study drug synergy in cancer [80, 81, 90] and to predict combinations of treatments to halt pro-angiogenesis activity of monocytes in breast cancer [91], for example. Due to this versatility and simplicity, logic models are promising tools to use for studying complex and heterogenous combination therapies.

No single approach is likely to be able to model with enough detail and at the same time scale up well enough to cover everything under consideration. For example, a logic model might be able to cover a large number of pathways in different cell types and the communication among these cells but not be able to precisely model the molecular mode of action of a drug; by contrast, a detailed dynamic mechanistic model can describe such molecular interactions in detail but will only be able to cover a few proteins within a cell. For this reason, hybrid strategies that combine different methodologies are likely to be needed to build such models. Indeed, such multiformalism models are becoming increasingly popular [92], and a range of approaches have been reported to link the macroscopic aspects of cancer, such as tumor growth, with the effects of specific therapies [87].

## Conclusions

Significant progress has been made in the identification of drugs to tackle tumor development by targeting tumor cell signaling that is driven by genetic aberrations, by alleviating protection from the tumor microenvironment, or by boosting the antitumor immune response. Most efforts in pre-clinical discovery of effective drug combinations, however, have focused only on the direct impact of drug combinations on signaling within a tumor cell. There is a significant opportunity to identify drug combinations that achieve a disproportionate benefit through an “accumulative efficacy”—that is, by optimally balancing effects on the heterogeneous cells of the tumor with effects on host cells and characteristics that collectively determine a patient’s outcome.

It is a potentially daunting prospect to consider generating the necessary data and computational approaches to model the fundamentally different nature of the effects of drugs on various cell types and system dynamics at the organism level. With recent advances in data generation platforms and computational approaches great strides have been made in this direction, although no single computational approach is likely to provide all the required aspects in enough detail and be able to scale up effectively. Knowledge-led formalisms can simulate the result of varying parameters and conditions that can be used to forecast the efficacy of therapies, but to provide useful personalized predictions they have to be able to simulate changes in all the key parameters that can be expected to influence the overall outcome of a patient. Data-driven approaches hold great promise to discover unforeseen relationships between drug effects and cell phenotypes, but they rely both on sufficient quantities of all the relevant data for training models and on incorporation of prior knowledge to overcome statistical limitations and redundancies in these data.

We advocate that more emphasis should be given to the generation of the necessary data and the development of the required computational approaches to model the full interplay between a therapy, a tumor, and the host. Knowledge-driven methodologies that are able to model the relationships between disparate data types and to report rationalized biological hypotheses will have a key role. Even then, it is likely that complementary experimental discovery platforms will be required alongside advanced preclinical models that recapitulate tumor–host interactions. Only through such intimate integration of experiments and computational modeling can we consider all the determinants of patient outcome and select optimal drug combinations.

## Box 1. Clinical trials of drug combinations in non-small-cell lung carcinoma

**Box 1.** Data were collected from Trialtrove [11] for non-small-cell lung carcinoma (NSCLC) trials published between 1996 and 2016 that tested multiple drugs and mentioned the word “combination” (or equivalent) in the description fields. Basket and umbrella trials that only tested drugs as monotherapies were excluded. Success rates reflect only the 521 trials that report a positive or negative outcome in Trialtrove; a further 1997 completed trials did not report an outcome and are not included in the graphs. A positive outcome is reported for trials that met their primary endpoint; however, the primary endpoint can vary and for this reason phase I trials (for which the endpoints were predominantly safety or pharmacodynamics) were separated from phase II, III, and IV trials (for which the endpoints were predominantly efficacy, response, or survival). Data include trials assessing combinations relative to respective monotherapies or relative to unrelated control arms.

Drugs are partitioned into one of five modes of action, which are detailed below:

- Tumor driver: the primary drug target is a protein within (or on the surface of) a cancer cell and drives a hallmark oncogenic process such as growth, survival, or repair
- Immune: the primary drug target is a protein within (or on the surface of) an immune cell or an immunosuppressive protein on the surface of a cancer cell
- Angiogenesis: the primary drug target is a protein that controls the development of tumor vasculature
- Classical cytotoxic: drugs that non-specifically target dividing or unstable cells
- Other: drugs that target processes that are outside of the tumor or unrelated to the disease, such as steroids, nutritional supplements, analgesics, or therapy associated with a comorbidity

The reported number of trials involving agents with each mode of action refers to the number of Trialtrove entries (independent trials) that involve one or more agents with a specific mode of action. Therefore, a trial involving multiple agents with the same mode of action will be counted only once for that mode of action, and a trial involving several agents with different modes of action may be counted for multiple modes of action.

From our analysis we can come to the following conclusions:

- A significant proportion of recent clinical trials testing drug combinations in NSCLC involve drugs that do not target cancer cells. Trials involving immune-targeting agents are mostly ongoing. Aside from tumor-targeting and immune-targeting agents, the proportions of drugs with other modes of action remain consistent between ongoing trials and those reporting negative or positive outcomes.
- A dramatic increase is apparent over recent years in the proportion of NSCLC trials of drug combinations involving immune-targeting agents. The proportion of trials involving drugs that target tumor drivers within cancer cells has been stable since 2007. The proportion of trials involving cytotoxic drugs continues to decrease.
- The 229 NSCLC trials with negative outcomes in Trialtrove show that, as expected, phase 1 trials are predominantly terminated owing to safety concerns or adverse effects for drugs that target the following mechanisms of action: cytotoxic (graph i), tumor cell driver targeting (graph ii), angiogenesis targeting (graph iii), immune targeting (graph iv), and other (graph v). By contrast, phase II, III, and IV trials are mostly terminated owing to lack of efficacy or the primary endpoint not being met. Proportionally fewer combination trials involving biologic (typically antibody-based) drugs report failures due to safety concerns or adverse effects (part vi), and since most immune-targeting agents are biologics the relative proportion of their trials terminated owing to lack of efficacy is increased (graph iv).

## Abbreviations

B_reg_ cell: Regulatory B cell
CAF: Cancer-associated fibroblast
CCLE: Cancer Cell Line Encyclopedia
CRISPR: Clustered regularly interspaced short palindromic repeats
DC: Dendritic cell
ECM: Extracellular matrix
ENCODE: The Encyclopedia of DNA Elements
GDSC: Genomics of Drug Sensitivity in Cancer
GEM: Genetically engineered mouse
GTEx: Genotype-Tissue Expression Project
ICGC: International Cancer Genome Consortium
LINCS: Library of Network-Based Cellular Signatures
MDSC: Myeloid-derived suppressor cell
NGS: Next-generation sequencing
NK: Natural killer
NSCLC: Non-small-cell lung carcinoma
PD-1: Programmed cell death protein 1
PD-L1: Programmed cell death protein 1 ligand 1
siRNA: Small interfering RNA
TCGA: The Cancer Genome Atlas
T_reg_ cell: Regulatory T cell

## References

[1] Holohan C, Van Schaeybroeck S, Longley DB, Johnston PG (2013). Cancer drug resistance: an evolving paradigm. Nat Rev Cancer.

[2] Al-Lazikani B, Banerji U, Workman P (2012). Combinatorial drug therapy for cancer in the post-genomic era. Nat Biotechnol.

[3] Chen S-H, Lahav G (2016). Two is better than one; toward a rational design of combinatorial therapy. Curr Opin Struct Biol.

[4] Bulusu KC, Guha R, Mason DJ, Lewis RPI, Muratov E, Motamedi YK (2016). Modelling of compound combination effects and applications to efficacy and toxicity: state-of-the-art, challenges and perspectives. Drug Discov Today.

[5] Lehár J, Krueger AS, Avery W, Heilbut AM, Johansen LM, Price ER (2009). Synergistic drug combinations tend to improve therapeutically relevant selectivity. Nat Biotechnol.

[6] O’Neil J, Benita Y, Feldman I, Chenard M, Roberts B, Liu Y (2016). An unbiased oncology compound screen to identify novel combination strategies. Mol Cancer Ther.

[7] Dietlein F, Kalb B, Jokic M, Noll EM, Strong A, Tharun L (2015). A synergistic interaction between Chk1- and MK2 inhibitors in *KRAS*-mutant cancer. Cell.

[8] Ryall KA, Tan AC (2015). Systems biology approaches for advancing the discovery of effective drug combinations. J Cheminform.

[9] Bansal M, Yang J, Karan C, Menden MP, Costello JC, Tang H (2014). A community computational challenge to predict the activity of pairs of compounds. Nat Biotechnol.

[10] Wu M, Sirota M, Butte AJ, Chen B. Characteristics of drug combination therapy in oncology by analyzing clinical trial data on ClinicalTrials.gov. Pac Sym Biocomput. 2015; doi:10.1142/9789814644730_0008. PMC4361221 25592569

[11] Trialtrove. Pharma Intelligence, New York. 2016. https://citeline.com/products/trialtrove/. Accessed 22 June 2016.

[12] Sharma P, Allison JP (2015). Immune checkpoint targeting in cancer therapy: toward combination strategies with curative potential. Cell.

[13] Puré E, Lo A (2016). Can targeting stroma pave the way to enhanced antitumor immunity and immunotherapy of solid tumors?. Cancer Immunol Res.

[14] Longo DL (2012). Tumor heterogeneity and personalized medicine. N Engl J Med.

[15] Tirosh I, Izar B, Prakadan SM, Wadsworth MH, Treacy D, Trombetta JJ (2016). Dissecting the multicellular ecosystem of metastatic melanoma by single-cell RNA-seq. Science.

[16] Thress KS, Paweletz CP, Felip E, Cho BC, Stetson D, Dougherty B (2015). Acquired *EGFR* C797S mutation mediates resistance to AZD9291 in non-small cell lung cancer harboring *EGFR* T790M. Nat Med.

[17] Zhao B, Pritchard JR, Lauffenburger DA, Hemann MT (2014). Addressing genetic tumor heterogeneity through computationally predictive combination therapy. Cancer Discov.

[18] Pritchard JR, Bruno PM, Gilbert LA, Capron KL, Lauffenburger DA, Hemann MT (2013). Defining principles of combination drug mechanisms of action. Proc Natl Acad Sci U S A.

[19] Zhao B, Sedlak JC, Srinivas R, Creixell P, Pritchard JR, Tidor B (2016). Exploiting temporal collateral sensitivity in tumor clonal evolution. Cell.

[20] Singh A, Settleman J (2010). EMT, cancer stem cells and drug resistance: an emerging axis of evil in the war on cancer. Oncogene.

[21] Lee MJ, Ye AS, Gardino AK, Heijink AM, Sorger PK, MacBeath G (2012). Sequential application of anticancer drugs enhances cell death by rewiring apoptotic signaling networks. Cell.

[22] Lopez JS, Banerji U (2016). Combine and conquer: challenges for targeted therapy combinations in early phase trials. Nat Rev Clin Oncol.

[23] O’Connor MJ (2015). Targeting the DNA damage response in cancer. Mol Cell.

[24] Paek AL, Liu JC, Loewer A, Forrester WC, Lahav G (2016). Cell-to-cell variation in p53 dynamics leads to fractional killing. Cell.

[25] Klemm F, Joyce JA (2015). Microenvironmental regulation of therapeutic response in cancer. Trends Cell Biol.

[26] Schmitz S, Machiels J-P (2016). Targeting the tumor environment in squamous cell carcinoma of the head and neck. Curr Treat Options Oncol.

[27] Ko C-J, Huang C-C, Lin H-Y, Juan C-P, Lan S-W, Shyu H-Y (2015). Androgen-induced TMPRSS2 activates matriptase and promotes extracellular matrix degradation, prostate cancer cell invasion, tumor growth, and metastasis. Cancer Res.

[28] Straussman R, Morikawa T, Shee K, Barzily-Rokni M, Qian ZR, Du J (2012). Tumour micro-environment elicits innate resistance to RAF inhibitors through HGF secretion. Nature.

[29] Overall CM, Kleifeld O (2006). Validating matrix metalloproteinases as drug targets and anti-targets for cancer therapy. Nat Rev Cancer.

[30] Khawar IA, Kim JH, Kuh H-J (2015). Improving drug delivery to solid tumors: priming the tumor microenvironment. J Control Release.

[31] Vasudev NS, Reynolds AR (2014). Anti-angiogenic therapy for cancer: current progress, unresolved questions and future directions. Angiogenesis.

[32] Mukaida N, Sasaki S (2016). Fibroblasts, an inconspicuous but essential player in colon cancer development and progression. World J Gastroenterol.

[33] Gao H (2016). Shaping tumor microenvironment for improving nanoparticles delivery. Curr Drug Metab.

[34] Davis ME, Chen ZG, Shin DM (2008). Nanoparticle therapeutics: an emerging treatment modality for cancer. Nat Rev Drug Discov.

[35] Lee JJ, Perera RM, Wang H, Wu D-C, Liu XS, Han S (2014). Stromal response to Hedgehog signaling restrains pancreatic cancer progression. Proc Natl Acad Sci U S A.

[36] Topalian SL, Drake CG, Pardoll DM (2015). Immune checkpoint blockade: a common denominator approach to cancer therapy. Cancer Cell.

[37] Yang Y (2015). Cancer immunotherapy: harnessing the immune system to battle cancer. J Clin Invest.

[38] Topalian SL, Taube JM, Anders RA, Pardoll DM (2016). Mechanism-driven biomarkers to guide immune checkpoint blockade in cancer therapy. Nat Rev Cancer.

[39] Wargo JA, Cooper ZA, Flaherty KT (2014). Universes collide: combining immunotherapy with targeted therapy for cancer. Cancer Discov.

[40] Dry JR, Pavey S, Pratilas CA, Harbron C, Runswick S, Hodgson D (2010). Transcriptional pathway signatures predict MEK addiction and response to selumetinib (AZD6244). Cancer Res.

[41] Ebert PJR, Cheung J, Yang Y, McNamara E, Hong R, Moskalenko M (2016). MAP kinase inhibition promotes T cell and anti-tumor activity in combination with PD-L1 checkpoint blockade. Immunity.

[42] Marshall G, Howard Z, Dry J, Fenton S, Heathcote D, Gray N (2011). Benefits of mTOR kinase targeting in oncology: pre-clinical evidence with AZD8055. Biochem Soc Trans.

[43] Lastwika KJ, Wilson W, Li QK, Norris J, Xu H, Ghazarian SR (2016). Control of PD-L1 expression by oncogenic activation of the AKT–mTOR pathway in non–small cell lung cancer. Cancer Res.

[44] Hukelmann JL, Anderson KE, Sinclair LV, Grzes KM, Murillo AB, Hawkins PT (2016). The cytotoxic T cell proteome and its shaping by the kinase mTOR. Nat Immunol.

[45] Chen P-L, Roh W, Reuben A, Cooper ZA, Spencer CN, Prieto PA (2016). Analysis of immune signatures in longitudinal tumor samples yields insight into biomarkers of response and mechanisms of resistance to immune checkpoint blockade. Cancer Discov.

[46] Ott PA, Hodi FS, Buchbinder EI (2015). Inhibition of immune checkpoints and vascular endothelial growth factor as combination therapy for metastatic melanoma: an overview of rationale, preclinical evidence, and initial clinical data. Front Oncol.

[47] Hodi FS, Lawrence D, Lezcano C, Wu X, Zhou J, Sasada T (2014). Bevacizumab plus ipilimumab in patients with metastatic melanoma. Cancer Immunol Res.

[48] Rizvi NA, Hellmann MD, Snyder A, Kvistborg P, Makarov V, Havel JJ (2015). Mutational landscape determines sensitivity to PD-1 blockade in non–small cell lung cancer. Science.

[49] Higuchi T, Flies DB, Marjon NA, Mantia-Smaldone G, Ronner L, Gimotty PA (2015). CTLA-4 blockade synergizes therapeutically with PARP inhibition in BRCA1-deficient ovarian cancer. Cancer Immunol Res.

[50] Rooks MG, Garrett WS (2016). Gut microbiota, metabolites and host immunity. Nat Rev Immunol.

[51] Newton R, Priyadharshini B, Turka LA (2016). Immunometabolism of regulatory T cells. Nat Immunol.

[52] Zitvogel L, Ayyoub M, Routy B, Kroemer G (2016). Microbiome and anticancer immunosurveillance. Cell.

[53] Viaud S, Daillère R, Boneca IG, Lepage P, Pittet MJ, Ghiringhelli F (2014). Harnessing the intestinal microbiome for optimal therapeutic immunomodulation. Cancer Res.

[54] Vétizou M, Pitt JM, Daillère R, Lepage P, Waldschmitt N, Flament C (2015). Anticancer immunotherapy by CTLA-4 blockade relies on the gut microbiota. Science.

[55] Pitt JM, Vétizou M, Waldschmitt N, Kroemer G, Chamaillard M, Boneca IG (2016). Fine-tuning cancer immunotherapy: optimizing the gut microbiome. Cancer Res.

[56] Cleeland CS, Allen JD, Roberts SA, Brell JM, Giralt SA, Khakoo AY (2012). Reducing the toxicity of cancer therapy: recognizing needs, taking action. Nat Rev Clin Oncol.

[57] Gangadhar TC, Vonderheide RH (2014). Mitigating the toxic effects of anticancer immunotherapy. Nat Rev Clin Oncol.

[58] Hu JX, Thomas CE, Brunak S (2016). Network biology concepts in complex disease comorbidities. Nat Rev Genet.

[59] Savage N (2015). Mobile data: made to measure. Nature.

[60] Bender E (2015). Big data in biomedicine. Nature.

[61] Menden M, Wang D, Guan Y, Michael Mason, Yu T, Jang IS, et al. The AstraZeneca-Sanger drug combination prediction challenge. 2016. https://www.synapse.org/#!Synapse:syn4231880. Accessed 1 Oct 2016

[62] Serra-Musach J, Mateo F, Capdevila-Busquets E, de Garibay GR, Zhang X, Guha R (2016). Cancer network activity associated with therapeutic response and synergism. Genome Med.

[63] De Wolf H, De Bondt A, Turner H, Göhlmann HWH (2016). Transcriptional characterization of compounds: lessons learned from the public LINCS data. Assay Drug Dev Technol.

[64] van de Wetering M, Francies HE, Francis JM, Bounova G, Iorio F, Pronk A (2015). Prospective derivation of a living organoid biobank of colorectal cancer patients. Cell.

[65] Li X, Nadauld L, Ootani A, Corney DC, Pai RK, Gevaert O (2014). Oncogenic transformation of diverse gastrointestinal tissues in primary organoid culture. Nat Med.

[66] Gao H, Korn JM, Ferretti S, Monahan JE, Wang Y, Singh M (2015). High-throughput screening using patient-derived tumor xenografts to predict clinical trial drug response. Nat Med.

[67] Delude CM (2015). Deep phenotyping: the details of disease. Nature.

[68] Hackl H, Charoentong P, Finotello F, Trajanoski Z (2016). Computational genomics tools for dissecting tumour–immune cell interactions. Nat Rev Genet.

[69] Angermueller C, Christof A, Tanel P, Leopold P, Oliver S (2016). Deep learning for computational biology. Mol Syst Biol.

[70] Lahat D, Dana L, Tulay A, Christian J (2015). Multimodal data fusion: an overview of methods, challenges, and prospects. Proc IEEE.

[71] Chen Y, Elenee Argentinis JD, Weber G (2016). IBM Watson: how cognitive computing can be applied to big data challenges in life sciences research. Clin Ther.

[72] You J (2015). Artificial intelligence. DARPA sets out to automate research. Science.

[73] Sun X, Bao J, Shao Y (2016). Mathematical modeling of therapy-induced cancer drug resistance: connecting cancer mechanisms to population survival rates. Sci Rep.

[74] Wilson S, Tod M, Ouerdani A, Emde A, Yarden Y, Adda Berkane A (2015). Modeling and predicting optimal treatment scheduling between the antiangiogenic drug sunitinib and irinotecan in preclinical settings. CPT Pharmacometrics Syst Pharmacol.

[75] Parra-Guillen ZP, Berraondo P, Ribba B, Trocóniz IF (2013). Modeling tumor response after combined administration of different immune-stimulatory agents. J Pharmacol Exp Ther.

[76] Gallo JM, Birtwistle MR (2015). Network pharmacodynamic models for customized cancer therapy. Wiley Interdiscip Rev Syst Biol Med.

[77] Sorger PK, Allerheiligen S. Quantitative and systems pharmacology in the post-genomic era: new approaches to discovering drugs and understanding therapeutic. NIH White Paper. 2011. https://www.nigms.nih.gov/training/documents/systemspharmawpsorger2011.pdf. Accessed 1 Oct 2016.

[78] Vicini P, van der Graaf PH (2013). Systems pharmacology for drug discovery and development: paradigm shift or flash in the pan?. Clin Pharmacol Ther.

[79] Saez-Rodriguez J, MacNamara A, Cook S (2015). Modeling signaling networks to advance new cancer therapies. Annu Rev Biomed Eng.

[80] Flobak Å, Baudot A, Remy E, Thommesen L, Thieffry D, Kuiper M (2015). Discovery of drug synergies in gastric cancer cells predicted by logical modeling. PLoS Comput Biol.

[81] Kirouac DC, Du JY, Lahdenranta J, Overland R, Yarar D, Paragas V (2013). Computational modeling of *ERBB2*-amplified breast cancer identifies combined ErbB2/3 blockade as superior to the combination of MEK and AKT inhibitors. Sci Signal.

[82] Fitzgerald JB, Schoeberl B, Nielsen UB, Sorger PK (2006). Systems biology and combination therapy in the quest for clinical efficacy. Nat Chem Biol.

[83] Korkut A, Wang W, Demir E, Aksoy BA, Jing X, Molinelli EJ (2015). Perturbation biology nominates upstream-downstream drug combinations in RAF inhibitor resistant melanoma cells. Elife.

[84] Yugi K, Kubota H, Hatano A, Kuroda S (2016). Trans-omics: how to reconstruct biochemical networks across multiple “omic” layers. Trends Biotechnol.

[85] Karr JR, Sanghvi JC, Macklin DN, Gutschow MV, Jacobs JM, Bolival B (2012). A whole-cell computational model predicts phenotype from genotype. Cell.

[86] Gonçalves E, Bucher J, Ryll A, Niklas J, Mauch K, Klamt S (2013). Bridging the layers: towards integration of signal transduction, regulation and metabolism into mathematical models. Mol Biosyst.

[87] Li XL, Oduola WO, Qian L, Dougherty ER (2015). Integrating multiscale modeling with drug effects for cancer treatment. Cancer Inform.

[88] Abou-Jaoudé W, Traynard P, Monteiro PT, Saez-Rodriguez J, Helikar T, Thieffry D (2016). Logical modeling and dynamical analysis of cellular networks. Front Genet.

[89] Fisher J, Piterman N, Bodik R (2014). Toward synthesizing executable models in biology. Front Bioeng Biotechnol.

[90] Morris MK, Clarke DC, Osimiri LC, Lauffenburger DA (2016). Systematic analysis of quantitative logic model ensembles predicts drug combination effects on cell signaling networks. CPT Pharmacometrics Syst Pharmacol.

[91] Guex N, Crespo I, Bron S, Ifticene-Treboux A, Faes-Van’t Hull E, Kharoubi S (2015). Angiogenic activity of breast cancer patients’ monocytes reverted by combined use of systems modeling and experimental approaches. PLoS Comput Biol.

[92] Stéphanou A, Volpert V (2015). Hybrid modelling in biology: a classification review. Math Model Nat Phenom.

[93] Iorio F, Knijnenburg TA, Vis DJ, Bignell GR, Menden MP, Schubert M (2016). A landscape of pharmacogenomic interactions in cancer. Cell.

[94] Barretina J, Caponigro G, Stransky N, Venkatesan K, Margolin AA, Kim S (2012). The Cancer Cell Line Encyclopedia enables predictive modelling of anticancer drug sensitivity. Nature.

[95] Lamb J, Crawford ED, Peck D, Modell JW, Blat IC, Wrobel MJ (2006). The Connectivity Map: using gene-expression signatures to connect small molecules, genes, and disease. Science.

[96] GTEx Consortium (2015). The Genotype-Tissue Expression (GTEx) pilot analysis: multitissue gene regulation in humans. Science.

[97] Uhlén M, Pontén F, Lindskog C (2015). Charting the human proteome: understanding disease using a tissue-based atlas. Science.

[98] The Cancer Genome Atlas Research Network (2013). The Cancer Genome Atlas pan-cancer analysis project. Nat Genet.

[99] The International Cancer Genome Consortium (2010). International network of cancer genome projects. Nature.

[100] ENCODE Project Consortium (2004). The ENCODE (ENCyclopedia Of DNA Elements) Project. Science.

